# Evaluating the discriminating capacity of cell death (apoptotic) biomarkers in sepsis

**DOI:** 10.1186/s40560-018-0341-5

**Published:** 2018-11-13

**Authors:** Christopher Duplessis, Michael Gregory, Kenneth Frey, Matthew Bell, Luu Truong, Kevin Schully, James Lawler, Raymond J. Langley, Stephen F. Kingsmore, Christopher W. Woods, Emanuel P. Rivers, Anja K. Jaehne, Eugenia B. Quackenbush, Vance G. Fowler, Ephraim L. Tsalik, Danielle Clark

**Affiliations:** 1 Biological Defense Research Directorate, Naval Medical Research Center, 503 Robert Grant Avenue, Silver Spring, MD 20910 USA; 2 Department of Pharmacology and Center for Lung Biology, University of South Alabama College of Medicine, Mobile, USA; 3 Rady Pediatric Genomic and Systems Medicine Institute, Rady Children’s Hospital, Encinitas, USA; 4 Division of Infectious Diseases and International Health, Department of Medicine, Duke University School of Medicine, Durham, USA; 5 Center for Applied Genomics and Precision Medicine, Department of Medicine, Duke University School of Medicine, Durham, USA; 6 Section on Infectious Diseases, Durham Veteran’s Affairs Medical Center, Durham, USA; 7 Department of Emergency Medicine, Henry Ford Hospital, Wayne State University, Detroit, USA; 8 Department of Emergency Medicine, University of North Carolina Health Care, Chapel Hill, USA; 9 Emergency Medicine Service, Durham Veteran’s Affairs Medical Center, Durham, USA

**Keywords:** Cell-free DNA, Nucleosomes, Severe Sepsis, Procalcitonin, Sepsis prognostication, Sepsis diagnosis

## Abstract

**Background:**

Sepsis biomarker panels that provide diagnostic and prognostic discrimination in sepsis patients would be transformative to patient care. We assessed the mortality prediction and diagnostic discriminatory accuracy of two biomarkers reflective of cell death (apoptosis), circulating cell-free DNA (cfDNA), and nucleosomes.

**Methods:**

The cfDNA and nucleosome levels were assayed in plasma samples acquired in patients admitted from four emergency departments with suspected sepsis. Subjects with non-infectious systemic inflammatory response syndrome (SIRS) served as controls. Samples were acquired at enrollment (T0) and 24 h later (T24). We assessed diagnostic (differentiating SIRS from sepsis) and prognostic (28-day mortality) predictive power. Models incorporating procalcitonin (diagnostic prediction) and APACHE II scores (mortality prediction) were generated.

**Results:**

Two hundred three subjects were included (107 provided procalcitonin measurements). Four subjects exhibited uncomplicated sepsis, 127 severe sepsis, 35 septic shock, and 24 had non-infectious SIRS. There were 190-survivors and 13 non-survivors. Mortality prediction models using cfDNA, nucleosomes, or APACHEII yielded AUC values of 0.61, 0.75, and 0.81, respectively. A model combining nucleosomes with the APACHE II score improved the AUC to 0.84. Diagnostic models distinguishing sepsis from SIRS using procalcitonin, cfDNA(T0), or nucleosomes(T0) yielded AUC values of 0.64, 0.65, and 0.63, respectively. The three parameter model yielded an AUC of 0.74.

**Conclusions:**

To our knowledge, this is the first head-to-head comparison of cfDNA and nucleosomes in diagnosing sepsis and predicting sepsis-related mortality. Both cfDNA and nucleosome concentrations demonstrated a modest ability to distinguish sepsis survivors and non-survivors and provided additive diagnostic predictive accuracy in differentiating sepsis from non-infectious SIRS when integrated into a diagnostic prediction model including PCT and APACHE II. A sepsis biomarker strategy incorporating measures of the apoptotic pathway may serve as an important component of a sepsis diagnostic and mortality prediction tool.

## Background

Sepsis remains a leading cause of mortality globally. Despite concerted research into improving treatment and survival, few novel efficacious therapies have been identified. Sepsis contributes up to 750,000 hospitalizations annually in the USA, is the most common etiology of ICU-associated mortality, and incurs 50% mortality rates in severe cases [1–7]. The pathophysiology of sepsis is complex, multifactorial, and heterogeneous involving multiple interdependent pathways (proinflammatory, anti-inflammatory, regulatory, and coagulation/fibrinolysis) which become dysregulated and uncoordinated [8].

Given the heterogeneity of sepsis, accurate diagnosis, stratification, and prognosis will require biomarker panels to capture the evolving and dynamic information provided by multiple unique and interdependent cascades [9, 10]. Sepsis biomarker panel candidates may require representation of (1) multiple non-collinear pathways (from a potentially infinite orthogonal space), (2) counter-regulatory biomarkers (capturing uncoordinated and dysregulated activity), and (3) temporal trends (“kinetics”) [11]. For example, procalcitonin (PCT) dynamics are superior in sepsis prognostication than isolated measurements [12]. Apoptosis is a process in which intracellular death programs are activated (programmed cell death). Apoptotic cells shrink and condense with a collapse of the cytoskeleton accompanied by dissemblance of the nuclear envelope and leakage of intact and degraded DNA fragments [3, 13].

The apoptosis pathway is increasingly recognized as integral to sepsis pathophysiology, and therefore, representative biomarkers may provide additive discriminatory power in sepsis biomarker panels [3, 13]. Apoptotic pathway activation increases with sepsis severity often leading to marked lymphopenia within the initial 24 h [14, 15]. Apoptotic depletion of immune cells can undermine host immunity by engendering anergy, latent infection reactivation, and susceptibility to secondary infections [4, 14, 15]. Apoptosis-induced cellular debris increases immunogenic cellular by-products (including damage-associated molecular patterns (DAMPs)) contributing to immune tolerance and deleterious immune activation and dysregulation [3, 4, 14–17]. As apoptotic biomarkers represent the integrated cumulative organ injury and systemic inflammation portending future tissue damage [13], they may provide independent, non-collinear (additive) discriminatory predictive power when combined with traditional biomarkers or the APACHEII score [3, 13, 15, 18]. Extracellular cell-free DNA (cfDNA) and nucleosome levels both reflect cellular apoptosis activity and may serve as representative biomarkers of this pathway [3, 19, 20].

Circulating cfDNA (encompassing nuclear and mitochondrial-DNA) derives from cellular necrosis, lysis, apoptosis, and secretion (i.e., neutrophil extracellular traps (NETs)) [3, 13, 21–23]. Although bacteria may contribute, it appears to be a minor contributor in infectious syndromes [3]. Nucleosomes are basic units of DNA packaging stemming from chromatin degradation by endonucleases during apoptosis or necrosis [24, 25]. Nucleosomes are the basic unit of DNA packaging whereby DNA is wound around histone core proteins. Histones represent a class of DAMP molecules, are cytotoxic to endothelial and epithelial cells, and contribute to NETosis, which also directly participates in inflammation and the response to infection (activating TLR2, TLR4, and NF-κβ signaling) [1, 26]. In healthy individuals, circulating cfDNA and nucleosome levels are low, exhibiting short half-lives (15 min) given efficient clearance (in the liver) [1, 22]. In illness, levels rise from excessive cellular injury/death, insufficient clearance, or decrements in endogenous DNase [1, 15, 24]. Improvement in detection has fostered studies in many clinical arenas (cancer, trauma, stroke, myocardial infarction, rheumatoid arthritis, and sepsis) to assess their utility as discriminative diagnostic and prognostic biomarkers [3, 15, 17, 20–22, 27]. Furthermore, pilot studies suggest cfDNA and nucleosome concentrations correlate with sepsis severity [15, 22]. The origins of both cfDNA and nucleosomes depend on the particular clinical syndrome. For example, extracellular nucleic acids are plausibly released from the direct injurious insult sustained in trauma, or the rapidly dividing tumor cells in cancer. In sepsis, it is thought these extracellular nucleic acids derive from hematologic cells (neutrophils and lymphocytes) participating in the immune response to infection, with contributions from tissue injury sustained in organ damage [3, 17, 23]. We are unaware of associations between cfDNA and nucleosome concentrations with preceding neutrophil and lymphocyte levels (i.e., immune-suppressed patients), nor attributable contributions from the hematological cells vice tissue injury across sepsis severity all warranting future study.

We measured cfDNA and nucleosome levels in archived plasma samples acquired from the Community Acquired Pneumonia and Sepsis Outcome Diagnostic (CAPSOD) study. Our primary objectives were to assess the diagnostic (differentiating SIRS and sepsis) and prognostic (28-day mortality) performance of both biomarkers. In this preliminary biomarker discovery effort, we included PCT and APACHE II score to determine if apoptotic biomarkers offered independent classification utility. Specifically, models incorporating procalcitonin (for diagnostic prediction) and APACHE II scores (for mortality prediction) were also generated.

## Methods

### Archived samples from the CAPSOD investigation

**Fig. 1:**
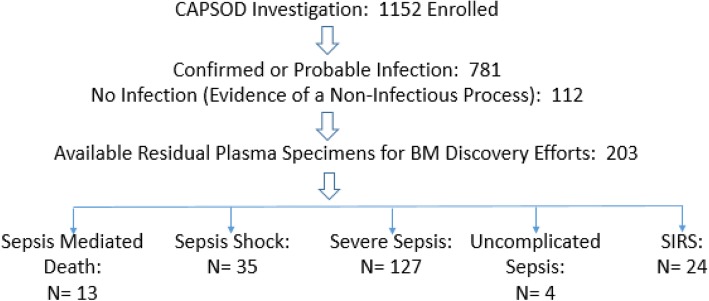
Enrollment flowchart

In the CAPSOD study (ClinicalTrials.gov NCT00258869), 1152 individuals with suspected community-acquired sepsis [≥ 2 Systemic Inflammatory Response Syndrome (SIRS) criteria presumed due to an infection] were enrolled prospectively in the emergency departments at three urban, tertiary-care hospitals in the USA (Duke University, Durham VA Medical Center, and Henry Ford Hospital) from 2005 to 2009 (Fig. 1). Later, a fourth emergency department was added (UNC Medical Center) where enrollment occurred in 2010. Some were later adjudicated as having non-infectious SIRS. Medical history, physical examination, and acute illness scores (APACHE II) were recorded at enrollment (T0) and 24 h later (T24). Blood specimens were acquired at the corresponding time-points [6].

The primary outcome was survival at day-28, which along with infectious status was adjudicated by board-certified clinicians. Definitions that were standard at that time were employed for organ dysfunction and shock [6]. The definitions used for the adjudication process were based on the 2001 Consensus definition for sepsis [28]. The investigators pursued proteomics and metabolomics on patient samples to identify novel signatures predicting sepsis-associated mortality and have published a parsimonious set of metabolites exhibiting excellent prognostication for sepsis-associated mortality [6].

We accessed a sub-sample (*n* = 203) of the enrolled patients in this effort. Subject selection from the pool of 1152 subjects was first constrained by removing indeterminate adjudications where individual subjects could not be definitively assigned as having sepsis or SIRS. Sample selection was further constrained by those subjects for whom an adequate volume of residual plasma remained. This resulted in 203 evaluable subjects. We did not identify an introduction of any significant systematic bias imposed by the two constraints in this subcohort analyzed in terms of demographics (age, gender), source of infection, infectious pathogen, or representation across the various sepsis categories save for uncomplicated sepsis. Sample processing was harmonized at all participating sites with immediate separation of plasma subsequently frozen at − 20 °C and underwent one freeze-thaw cycle prior to assaying. This retrospective analysis of previously acquired specimens was approved by the Naval Medical Research Center (NMRC) institutional review board (IRB) as exempt (non-human subjects research) under protocol NRMC.2014.0008.

### Assays

#### cfDNA

The cfDNA was assayed using SYBR® Gold Nucleic Acid Gel Stain, (Invitrogen, Paisley, UK) via the fluorometric method [3]. This assay yields similar cfDNA levels employing serum or plasma, correlates significantly with conventional β-globin gene DNA quantification [*R*^2^  =  0.9987 (*p* < 0.0001)], and remains immune to organic molecular interference when samples are diluted to < 30% [3]. We executed the assay with slight modification (improving assay resolution). Specifically, SYBR® Gold was diluted at 1:1000 in dimethyl sulphoxide (DMSO, Sigma-Aldrich) and then at 1:8 in phosphate-buffered saline. An eight-point standard curve was created employing ultrapure salmon sperm DNA (Life Technologies, Carlsbad, CA, USA) diluted in 2% BSA/100 mM HEPES from 4 to 0.13 μg/ml [yielding a robust linear curve (*R*^2^ > 0.99) between 0.25 and 4 μg/ml] (corresponding to a sample range: 20 to 1.25 μg/ml upon 1:5 dilution in the assay) aligning with 1:5 dilutions (in 2% BSA/100 mM HEPES) applied to samples and controls [10 μg/ml and 5 μg/ml (2 μg/ml and 1 μg/ml upon 1:5 dilutions)]. A common plasma control was applied to all plates to control for plate-to-plate variability. Twenty microliters of standards, controls, and samples were applied to black 96-well plates (Greiner Bio-One, Frickenhausen, Germany). Diluted SYBR® Gold was added (80 μl) to each well (final dilution 1:10,000), and fluorescence measured with a 96-well fluorometer (Ultra Evolution; Tecan, Durham, NC, USA) at an emission wavelength of 535-nm and an excitation wavelength of 485-nm. The low end of our linear curve overlaps the anticipated upper end of the normal range of healthy patients (1 μg/ml) which is known to exhibit significant intra- and inter-individual variability [3]. The intra-day CV is 16%, 7.9%, and 4.8% and inter-day CV is 31%, 6.7%, and 8% in the low (383 ng/ml), elevated (1152 ng/ml), and high DNA range (2735 ng/mL), respectively [3].

##### Nucleosomes

The nucleosomes were quantified using the Cell Death Detection ELISAPlus kit (Roche Life Science, Indianpolis, IN, USA) according to the manufacturer’s instructions. This assay employs two murine antibodies directed at DNA (detection) and histones (capture). An eight-point standard curve was generated by twofold standard dilution of purified human nucleosomes (Human native nucleosomes; EMD Millipore, Billerica, MA, USA) in the incubation buffer from the enzyme-linked immunosorbent assay (ELISA) kit. Samples (18 uL) were assayed in duplicate. Standards and samples were applied followed by 80 uL of immunoreagent. Plates were incubated at (21 °C) for 2 h while shaking gently (300 rpm). Plates were decanted and washed thrice using 250 uL of incubation buffer/well. ABTS (100 uL) solution was added per well and incubated at room temperature with gentle shaking (250 rpm) for 15 min. The detection reaction was stopped with 100 uL of stop solution/well. The optical density of the wells at 405 nm were read on an Epoch microplate spectrophotometer (BioTek; Winooski, VT, USA). Standard curves were fitted to a five-parameter logistic curve using the GEN5 Data Analysis Software version 2.01 (BioTek) allowing interpolation for sample concentrations.

##### Procalcitonin

Procalcitonin (PCT) was measured from serum samples on a Roche Elecsys 2010 analyzer (Roche Diagnostics) by electrochemiluminescence or on the miniVIDAS immunoassay (bioMerieux). When serum was unavailable, measurements were made by the Phadia Immunology Reference Laboratory in plasma-EDTA by immunofluorescence using the BRAHMS PCT sensitive KRYPTOR (Thermo Fisher Scientific). Replicates were performed for some paired serum and plasma samples, revealing equivalence in concentrations. Therefore, all PCT measurements (ng/ml) were treated equivalently, regardless of testing platforms.

### Statistical analyses

Demographic and clinical data were compared with chi-squared, Student’s *t* test, or Wilcoxon rank sum test. Non-normal data were log-transformed. Spearman’s rank-order correlation coefficients were calculated to evaluate correlations between biomarkers and APACHE II scores. Statistical significance was defined as *p* < 0.05. Prediction of 28-day mortality and discrimination between SIRS and sepsis were performed using logistic regression models. Performance was evaluated using area under the curve (AUC). All analyses were done with Stata (version-14).

## Results

### Study population

This was a nested case-control study focusing on subjects within the CAPSOD cohort. After identifying individuals with residual plasma, we identified 203 subjects with clinically adjudicated sepsis (*n* = 179) or non-infectious SIRS (*n* = 24) (Table 2). The sepsis group was further stratified by sepsis severity using definitions available during the enrollment period (i.e., before Sepsis-3) and as previously defined [6]. The cohort was further stratified by 28-day survival. Overall mortality was low (6.4% mirroring the 4.9% in the full CAPSOD cohort) resulting in 190 survivors and 13 non-survivors. All the mortality events were in subjects with sepsis. Although the original investigation enrolled from four sites, most of our samples were derived from a single site (Duke). There were no significant differences in age, gender, or race between survivors and non-survivors. We did observe a significant difference in comorbidities. Non-survivors had a higher prevalence of cirrhosis, chronic kidney disease, and chronic pulmonary disease. Bacterial pathogens were recovered from 28% of subjects. *Staphylococcus aureus* was most common (*n* = 16) followed by *Escherichia coli* (*n* = 10), *Klebsiella pneumoniae* (*n* = 9), and *Streptococcus pneumoniae* (*n* = 7). Influenza A was identified from two patients: one survivor and one non-survivor (Table 1).

**Table 1:** Demographic table

Characteristic	Died (*N* = 13)	Survived (*N* = 190)
Age [median (IQR)]	64 (53–76)	54 (40–67)
Male [*n* (%)]	8 (62)	107 (56)
Caucasian [*n* (%)]	11 (85)	116 (61)
Comorbidities [*n* (%)] ^+^	9 (69)*	67 (35)*
CAP [*n* (%)]	7 (54)*	29 (15)*
Pathogen [*n* (%)]		
Unidentified	7 (54)	139 (73)
*Staphylococcus aureus*	1 (8)	15 (8)
*Escherichia coli*	1 (8)	9 (5)
*Klebsiella pneumoniae*	0 (0)	9 (5)
*Streptococcus pneumoniae*	1 (8)	6 (3)
Other	3 (23)	12 (6)

*Significant difference between survivors and non-survivors (*p* < 0.05)

^+^Comorbidities include liver failure, heart failure, renal failure, neoplasm, chronic lung disease, immunosuppression, neutropenia, HIV, hemodialysis, corticosteroid use, or chemotherapy

*CAP* community-acquired pneumonia

### Assessment of significance across sepsis stratifications in biomarkers (cfDNA, nucleosome, and procalcitonin concentrations) and APACHE II score (Table 2)

Under the hypothesis that apoptosis is a prominent host pathway in response to infection, we systematically compared cfDNA and nucleosome concentrations (seeking significant differences) among patients exhibiting various categories of sepsis (Table 2). Saliently, the sepsis severity categories delineated in Table 2 are based on the maximum severity achieved. Our subsequent analyses included comparing sepsis as compared to non-infectious SIRS, sepsis severities (i.e., uncomplicated sepsis, severe sepsis, and septic shock), and sepsis survival outcomes. Moreover, we compared concentrations at the time of enrollment (T0) and 24 h later (T24). Our reporting framework will assess cfDNA, followed by nucleosomes, PCT, and the APACHE II score.

**Table 2:** Mean biomarker concentrations and APACHE II scores at enrollment (T0) stratified by sepsis categories (maximum sepsis severity achieved)

	cfDNA (μg/ml) Mean (SD)^6^	Nucleosome (μg/ml) Mean (SD)^7^	APACHE II Mean (SD)^9^	^5,8^PCT (ng/ml) Mean (SD)
Non-infectious SIRS^1^ (*N* = 24)	3.0 (1.5)	1.1 (1.7)	8.3 (4.7)	7.5 (24.8)
Uncomplicated sepsis^2^ (*N* = 4)	3.6 (1.0)	1.7 (1.9)	11.5 (3.8)	0.4 (0.4)
Severe sepsis^3^ (*N* = 127)	3.9 (4.3)	3.0 (9.4)	9.0 (5.3)	6.5 (15.6)
Shock^4^ (*N* = 35)	4.8 (5.8)	5.5 (10.9)	15.5 (6.3)	20.7 (34.4)
Survivors (*N* = 190)	3.9 (4.3)	3.2 (9.1)	10.1 (5.9)	9.1 (21.9)
Non-survivors *(N* = 13)	3.9 (1.4)	5.0 (4.9)	17.5 (5.9)	–

^1^SIRS (no infection with two or more of the following): 1 temp > 38.3 or < 36 °C, 2 heart rate > 90 bpm, 3 tachypnea resp. > 20 bpm or pCO2 < 32 mmHg, 4 WBC < 4000mm^3^ or > 12,000mm^3^ or 10% bands

^2^Sepsis (infection with two or more of the aforementioned SIRS criteria)

^3^Severe sepsis: sepsis accompanied by organ dysfunction: 1 arterial hypotension (SAP < 90 mmHg, MAP < 70 mmHg), 2 reduced urine output (< 0.5 mL/kg/h for > 2 h); 3 acute lung injury (PaO_2_/FIO_2_ < 250 if without pneumonia or < 200 if afflicted with pneumonia) based on the ACCP/SCCM consensus document. The following organ systems surveillance was available: cardiovascular, pulmonary, renal, and hematologic

^4^Septic shock: sepsis-induced hypotension persisting despite adequate fluid resuscitation

^5^There were only three subjects available in the non-survivor group in which the PCT level was available

^6^Significance between SIRS and sepsis (*p* = 0.009), SIRS and severe sepsis (*p* = 0.02), SIRS and septic shock (*p* = 0.01), and SIRS and death (*p* = 0.04)

No significance between survivors and non-survivors

^7^Significance between SIRS and septic shock (*p* = 0.012); between SIRS and death (*p* = 0.001); between severe sepsis and death (*p* = 0.003); between sepsis vice SIRS (*p* = 0.036); between survivors and non-survivors (*p* = 0.007)

^8^Significance between SIRS and septic shock (*p* = 0.002); SIRS and severe sepsis (*p* < 0.002)

^9^Significance between SIRS and septic shock (*p* < 0.001); between SIRS and death (*p* < 0.001); between severe sepsis and septic shock and death (*p* < 0.001); between SIRS and sepsis (*p* = 0.044); between survivors and non-survivors (*p* < 0.001)

For the cfDNA evaluation, as expected, we observed a trend toward higher cfDNA concentrations as sepsis severity increased. Specifically, we noted significant differences (increases) in the mean cfDNA concentrations between SIRS patients (3.0 μg/ml) when compared to patients experiencing severe sepsis (3.9 μg/ml; *p* = 0.02), septic shock (4.8 μg/ml; *p* = 0.01), and death (3.9 μg/ml; *p* = 0.04). There was a significant difference in the cfDNA concentrations between subjects experiencing sepsis vice SIRS (*p* = 0.009). We did not identify a significant difference in the cfDNA levels between survivors and non-survivors.

With respect to nucleosomes, there were significant differences observed in the mean nucleosome concentrations between SIRS patients (1.1 μg/ml) when compared to patients experiencing septic shock (5.5 μg/ml; *p* = 0.012) and those who died (5.0 μg/ml; *p* = 0.001). We also noted significant differences between patients experiencing severe sepsis (3.0 μg/ml) and death (*p* = 0.003). There was a significant difference in the nucleosome concentrations between subjects experiencing sepsis vice SIRS (*p* = 0.036). We noted a significant difference in nucleosome concentrations between survivors and non-survivors (*p* = 0.007).

With respect to PCT, there were significant differences observed in the mean PCT concentration between patients experiencing SIRS (7.5 μg/ml) and septic shock (20.7 μg/ml; *p* = 0.002). We did observe a significant decrement in PCT levels from SIRS to severe sepsis (6.5 μg/ml; *p* < 0.002). There was no significant difference in PCT concentrations in subjects experiencing sepsis vice SIRS. There were too few mortality cases with PCT measurements for meaningful interpretation in mortality prediction models nor to assert differences between survivors and non-survivors.

The average APACHE II score was significantly lower in SIRS patients (8.3) when compared to patients experiencing septic shock (15.5; *p* < 0.001) or those who died (17.5; *p* < 0.001). APACHE II was also lower in patients experiencing severe sepsis (9.0) compared to septic shock and death (*p* < 0.001 for both comparisons). The APACHE II score was significantly elevated in subjects experiencing sepsis vice SIRS (*p* = 0.044). The APACHE II score was significantly higher in non-survivors vice survivors (*p* < 0.001).

**Fig. 2:**
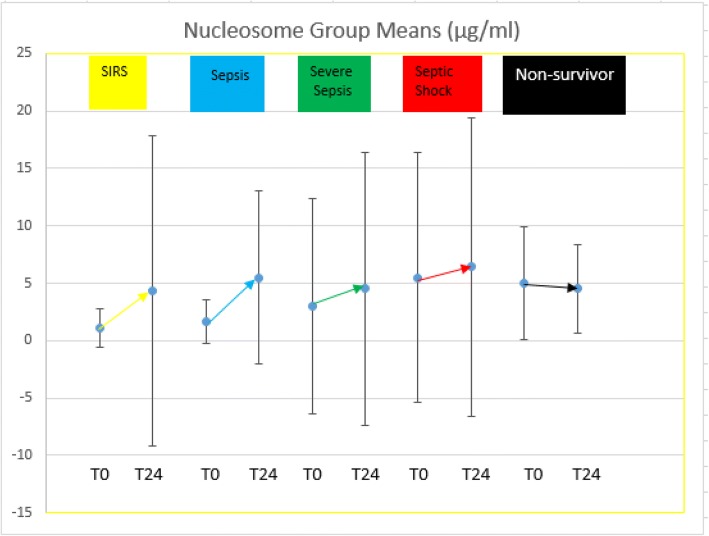
Nucleosome group means and standard deviations at T0 and T24. Gold (SIRS); blue (sepsis); green (severe sepsis); red (septic shock); black (non-survivors)

**Fig. 3:**
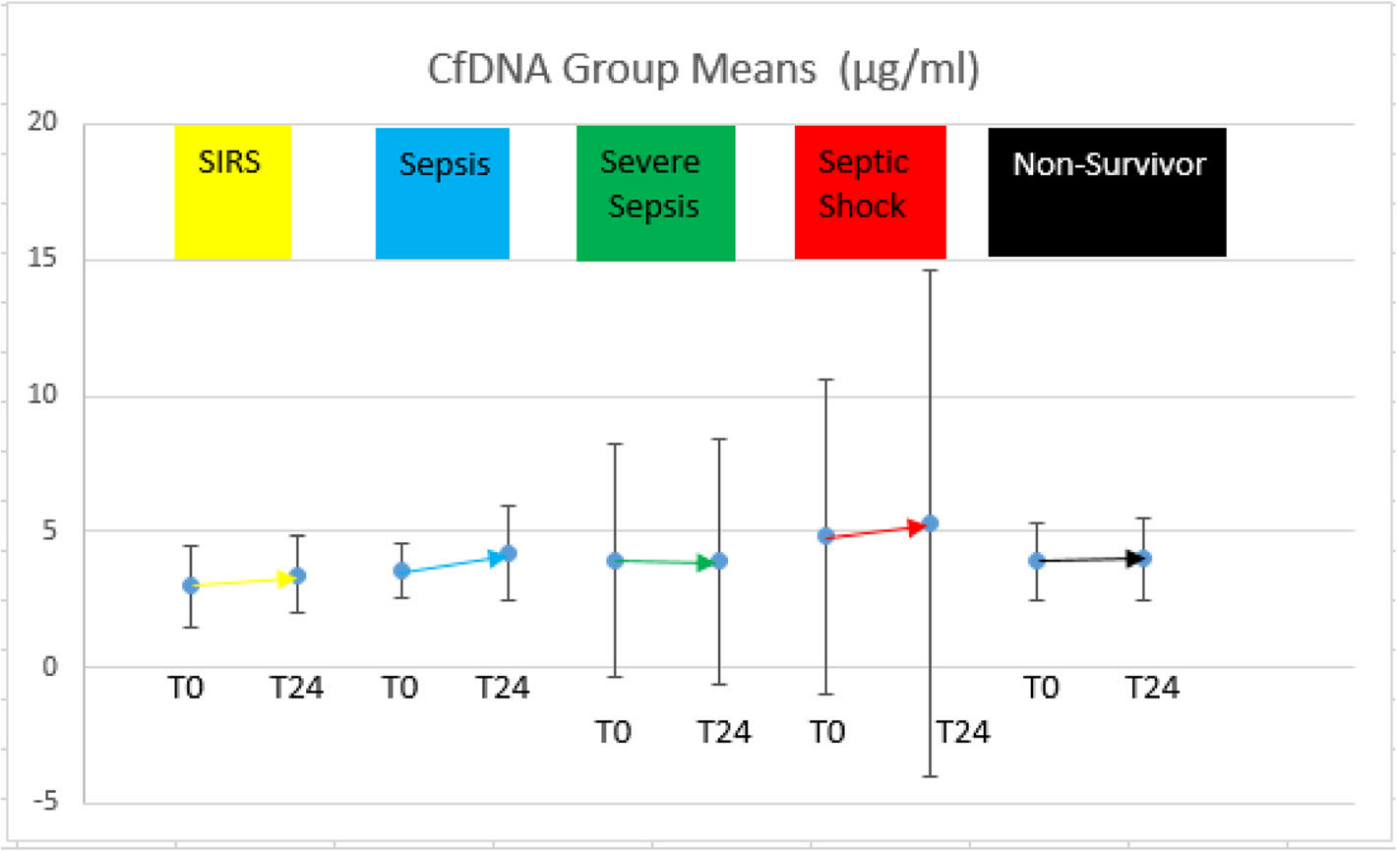
cfDNA group means and standard deviations at T0 and T24. Gold (SIRS); blue (sepsis); green (severe sepsis); red (septic shock); black (non-survivors)

Finally, we identified no significant difference in the cfDNA biomarker concentrations measured at T0 and T24. Unlike the cfDNA levels, we did identify significant elevations in the nucleosome levels from T0 to T24, accompanied by increased variability at T24 relative to cfDNA in all groups (SIRS, death, and all severities of sepsis). Given the increased variability and the fact that there was no difference in the predictive accuracy when exploiting nucleosomes at T0 vice T24, we present the concentrations for nucleosomes (and all biomarkers) at T0 in Table 2 and all analyses assessing predictive accuracy utilizes values at T0 (see Figs. 2 and 3).

### Biomarker correlations

Even though cfDNA and nucleosomes are both apoptotic biomarkers, they may reflect different aspects of that process. We therefore correlated concentrations of these two biomarkers with each other. Furthermore, these two apoptotic biomarkers may be co-linear or complementary with other prognostic biomarkers, specifically PCT and APACHE II score. We therefore also assessed correlations between cfDNA, nucleosomes, PCT, and APACHE II (Table 3). While there was a modest correlation between cfDNA and nucleosomes (0.41), there was a smaller correlation between cfDNA and PCT and APACHE II (0.29 and 0.21, respectively). The nucleosome levels exhibited a similarly low correlation with PCT and APACHE II (0.07 and 0.24, respectively).

**Table 3:** Correlation table

	cfDNA	Nucleosome	PCT^2^	APACHE II
cfDNA	1	0.41	0.29	0.21
Nucleosome		1	0.07	0.24
PCT^1^			1	0.41
APACHE II				1

Spearman’s correlation coefficient

^1^PCT concentrations available for 107 patients

### Predicting 28-day mortality

Mortality prediction models using cfDNA (at T0), nucleosomes (at T0), or APACHE II (at T0) yielded AUC values of 0.61, 0.75, and 0.81, respectively (Table 4). As asserted in Table 2, there were too few subjects in our cohort possessing PCT levels to provide meaningful interpretation. Permutations of a mortality prediction tool that used various combinations of cfDNA, nucleosomes, and APACHE II revealed that only nucleosome concentrations added to the predictive accuracy of APACHE II alone (0.84).

**Table 4:** AUC for predicting mortality

AUC for predicting mortality	
Biomarker or APACHE II Scoring System	AUC (*N* = 203) predicting mortality
cfDNA (T0)	0.61 (0.46–0.75)
cfDNA (T24)	0.62 (0.47–0.76)
Nucleosome (T0)	0.75 (0.62–0.87)
Nucleosome (T24)	0.67 (0.52–0.81)
APACHE II (T0)	0.81 (0.69–0.93)
APACHE II (T0) + cfDNA (T0)	0.81 (0.70–0.93)
APACHE II (T0) + nucleosome (T0)	0.84 (0.72–0.96)

### Discriminating SIRS from sepsis

Procalcitonin has been widely used as a sepsis diagnostic biomarker. We therefore determined whether measuring cfDNA or nucleosome improved the ability of procalcitonin to discriminate sepsis from SIRS. When used alone, we observed similarly modest AUCs for both cfDNA (0.62) and nucleosomes (0.63). When restricting to the subset of subjects who had available PCT results (*n* = 107), the AUC values for sepsis vs. SIRS discrimination were 0.64 for procalcitonin, 0.65 for cfDNA (at T0), and 0.63 for nucleosomes (at T0). There was no significant difference in the AUC for cfDNA and nucleosomes in the entire data set relative to the subset providing procalcitonin. A model incorporating all three biomarkers had an improved AUC of 0.74 (Table 5).

**Table 5:** AUC for diagnosis (differentiating SIRS from sepsis)

	AUC (*N* = 203) Predicting sepsis
cfDNA (T0)	0.62 (0.50–0.74)
Nucleosome (T0)	0.63 (0.52–0.73)
Subset of patients (*N* = 107) possessing PCT values	
	AUC (*N* = 107) Predicting sepsis
Nucleosome (T0)	0.63 (0.46–0.79)
CfDNA (T0)	0.65 (0.44–0.85)
PCT (T0)	0.64 (0.49–0.79)
PCT (T0) + nucleosome (T0) + cfDNA (T0)	0.74 (0.60–0.88)

## Discussion

Apoptosis is a well-recognized biological pathway in the host’s response to infection [3, 13]. It is therefore plausible that cfDNA and nucleosomes, both by-products of the apoptosis pathway exhibiting biological roles (primarily originating from lymphocytes and neutrophils), could serve as useful sepsis biomarkers [29]. These biomarkers are inextricably linked within the interdependent innate and adaptive immunity (including NETosis), modulating endothelial homeostasis, and biasing the delicate balance within several pathways and their counter-regulatory cascades (including inflammatory, coagulation, and fibrinolytic) [1, 26, 30]. It is not yet known if altering cfDNA or nucleosome levels or their dynamics could influence the evolution and outcomes in sepsis. In this study, we sought to answer a more proximal question, which is whether cfDNA or nucleosomes correlated with various aspects of sepsis such as diagnosis, severity, and prognosis. Furthermore, we wished to assess how these biomarkers compared with each other and what importance might they serve when combined with other sepsis-related biomarkers and predictive scores. As prior studies suggested that the cfDNA level in sepsis patients are primarily host derived (nuclear) in origin (not secondary to prokaryotic origins), we did not pursue a rigorous delineation of the source of nucleic acid in this effort as the major impetus was to seek a clinically relevant and reliable predictive diagnostic and prognostic biomarker panel [3, 13].

The prognostic accuracy of cfDNA to predict sepsis-mediated mortality was modest in this study (AUC of 0.61) but is comparable to that reported in the literature for non-ICU admitted subjects exhibiting AUCs (0.61 to 0.84) [3, 17, 22]. Notably, hitherto enrollment has been restricted to severely septic patients admitted to the ICU wherein the cfDNA exhibited superior prognostic accuracy (AUC of 0.7–0.97) [3, 13, 15, 17, 22, 27]. The lower accuracy observed in our investigation may be attributed to the (1) small sample size (and most saliently few subjects experiencing mortality), (2) inclusion of less ill sepsis patients irrespective of ICU admission, (3) possible sample degradation from long-term storage (although speculative should have been minimized exploiting our chosen assay), (4) the pronounced intra and inter-individual variability in cfDNA levels, (5) differential renal clearance, and (6) differential comorbidities that likely contribute to poorer predictive accuracy in patients experiencing less severe sepsis (and non-infectious SIRS). Of note, we had too few cases of specific clinical syndromes (comorbidities) and most specifically renal insufficiency to control for and evaluate their independent influence upon mortality and certainly warrants attention in future investigations. *Thus, cfDNA levels may be best exploited in severe sepsis patients admitted to the ICU*. Finally, of note, there have been no significant differences in cfDNA levels observed relative to the type of infecting organism [31].

The prognostic accuracy of nucleosomes for predicting sepsis-associated mortality was higher than that predicted by cfDNA and more consistent with published literature [14, 19]. We observed a modest correlation between these two biomarkers, which was less than expected given their shared biology. We speculate this may attributed to (1) differential nucleosome and cfDNA levels generated via variable apoptosis/necrosis ratios and NETosis, (2) cfDNA encompassing non-nuclear (mtDNA), (3) differential half-lives and clearance kinetics of their components, and (4) variability introduced by the assay method (ELISA) [1]. These observations suggest that in addition to concerted attention to consider potential representation in predictive biomarker panels, multiple apoptotic biomarkers (cfDNA, mucleosomes, histones) may be considered.

Both cfDNA and nucleosome (the latter significantly) were higher in non-survivors, but neither biomarker discriminated sepsis severity among survivors consistent with published data [3, 17, 18, 21, 22, 27, 32–35]. Increased levels among non-survivors may stem from a discrete increase in immune cell destruction, and bias to necrosis relative to caspase-dependent apoptosis in non-survivors [1, 32]. This suggests a reproducible dichotomy in host molecular responses highlighting allostasis (pathway normalization or compensation) in survivors and maladaption (pathway dysregulation and funneling to conserved death pathways) in non-survivors.

PCT is a host response biomarker that is secreted primarily in the context of bacterial infection but can also be elevated in certain non-infectious conditions. However, it is frequently used to help discriminate sepsis from non-infectious conditions. Moreover, it is part of the host’s inflammatory pathway and presumably represents biology that is largely orthogonal to apoptosis. The subjects from which PCT was acquired may have experienced clinical endpoints deviating systematically from the entire cohort. However, we did not identify any systematic differences in demographics or salient clinical parameters between these cohorts, as delineated in Table 1. APACHE II is a commonly used clinical score that incorporates a variety of host factors such as age, comorbidity, and organ function assessments to create a sepsis severity score which correlates with mortality. Regrettably, as intimated earlier, the CAPSOD investigation experienced a lower percentage of non-survivors than historical or contemporary reported sepsis-associated mortality rates. Thus, we had too few non-survivors possessing PCT levels to pursue a mortality prediction model. Given the poor correlation of these apoptotic biomarkers with both PCT and the APACHE II, and the known biology of apoptosis, we conjecture that apoptotic biomarkers reflect an important septic pathway non-collinear or not otherwise reflected in the APACHE II or other biomarkers (PCT) and thus would be complimentary to them, thus adding accuracy to the mortality prediction model [3, 13]. We observed a modest increase in the AUC (from 0.81 to 0.84) appending nucleosomes to the APACHE II which although unlikely to add clinically significant predictive prognostic discrimination, we suspect that the additive predictive accuracy of the apoptotic biomarkers was muted by the unusually high predictive value of the APACHE II score in this cohort secondary to the low numbers of non-survivors.

The rates of non-infectious etiologies misdiagnosed as sepsis are estimated to be 14–18% in the emergency department population [36]. Most biomarkers studied to date are insensitive in differentiating SIRS stemming from infectious or non-infectious etiologies [21]. Improvements in predictive diagnostic accuracy would expedite accurate diagnoses, promoting prompt and appropriate therapeutic intervention and circumventing unnecessary antibiotic exposure. Both apoptotic biomarkers exhibited poor diagnostic accuracy to differentiate SIRS from sepsis (we speculate due to dilution with non-ICU admitted patients), yet was consistent with prior literature (nucleosomes exhibited a diagnostic AUC for discriminating sepsis in ICU patients of 0.7 [4]). However, we did observe a significant increase in AUC (0.74) exploiting our three-parameter model demonstrating independent and additive diagnostic predictive power from the apoptosis pathways.

Serial testing of sepsis biomarkers (e.g., procalcitonin, caspase, cleaved cytokeratin18, and protein C) may provide superior discriminatory power [3, 37]. However, cfDNA may not be useful for serial monitoring since concentrations remain stable for several days following sepsis presentation suggesting a fixed burden of cumulative tissue injury dictated by sepsis severity [3, 13, 17, 37]. We corroborated these observations identifying nonsignificant differences in the cfDNA levels in our investigation at T0 and T24. We acknowledge that the “stationary” kinetics encompasses a dynamic of fluctuating nucleic acid derived from NETosis, apoptosis, necrosis, and endogenous DNase activity. Unlike the cfDNA levels, we did identify significant elevation in the nucleosome levels from T0 to T24 (accompanied by increased variability). However, we did not identify differential predictive value for diagnosis or prognosis at T24 from T0 (likely attributed to the wide variability). We are not aware of any literature describing nucleosome kinetics; thus, this is the first report revealing the increasing nucleosome concentrations in septic patients in the first 24 h, which deviates from the stationary kinetics exhibited by cfDNA. Given the relative dearth of research specific to nucleosomes in sepsis, further research is necessary to define its kinetics and variability as a function of clinical state. Whereas biomarker dynamics provide useful information about a patient’s state of illness and response to treatment, stable biomarkers can be useful clinically as they may provide a reliable inference as to the severity of sepsis at presentation (regardless of its heterogeneity), although not informing changing clinical states or treatment response.

Technology will need to be developed potentiating real-time measurement of cfDNA or nucleosomes so their relevance to clinical practice may be realized. Future research may attempt partitioning the source of cfDNA (mitochondrial, nuclear) and clarifying the predictive accuracy of the constituents of nucleosomes (histones) which may provide further insight into differentiating their relative propensity to promoting inflammation, coagulation, anti-fibrinolysis, antibacterial activity, and predictive (diagnostic and prognostication) power [2]. Alternative apoptotic biomarkers may be superior in their discriminatory potential and targeted for future research [31, 38]. Finally, all apoptotic biomarkers would ideally be developed as a real-time point of care testing platform.

### Limitations

We employed a convenience sampling constrained by subjects with definitive adjudicated diagnoses and who had sufficient banked plasma for biomarker measurements (although did not observe any significant systematic bias from the full cohort in terms of demographics (age, gender), source of infection, infectious pathogen, or representation across the various sepsis categories save for uncomplicated sepsis). The low mortality in this cohort may have resulted in a lower sensitivity of these biomarkers for severe disease. However, this did represent a more realistic cohort of patients with sepsis, not all of whom are managed in the ICU. Long-term storage may have led to differential cfDNA and nucleosome degradation which is uncontrolled with the retrospective analyses employed herein (however, we acknowledge the cfDNA fluorometric assay employed circumvents concerns in DNA fragmentation) [3]. Finally, samples underwent one freeze-thaw cycle, however, again the fluorometric assay circumvents concerns in DNA fragmentation, while nucleosome concentrations were shown to remain stable through several freeze-thaw cycles [25].

## Conclusions

To our knowledge, this is the first head-to-head comparison of cfDNA and nucleosomes in diagnosing sepsis and predicting sepsis-related mortality. Both cfDNA and nucleosome concentrations demonstrated a modest ability to distinguish sepsis survivors and non-survivors and provided additive diagnostic predictive accuracy in differentiating sepsis from non-infectious SIRS when integrated into a diagnostic prediction model including PCT and APACHE II. A sepsis biomarker strategy incorporating measures of the apoptotic pathway may serve as an important component of a sepsis diagnostic and mortality prediction tool.

## Abbreviations

AUC: Area under the curve
BSA: Bovine serum A
CAP: Community-acquired pneumonia
CAPSOD: Community Acquired Pneumonia and Sepsis Outcome Diagnostic
cfDNA: Cell-free DNA
DAMP: Damage-associated molecular pattern
ELISA: Enzyme-linked immunosorbent assay
NMRC: Naval Medical Research Center
PCT: Procalcitonin
SIRS: Systemic Inflammatory Response Syndrome
T0: Lab acquisition upon initial enrollment
T24: Lab acquisition at 24 h later
